# Inflammatory markers may mediate the relationship between processed meat consumption and metabolic unhealthy obesity in women: a cross sectional study

**DOI:** 10.1038/s41598-023-35034-6

**Published:** 2023-06-07

**Authors:** Azam Mohamadi, Farideh Shiraseb, Atieh Mirzababaei, Ahmad Mujtaba Barekzai, Cain C. T. Clark, Yasaman Aali, Khadijeh Mirzaei

**Affiliations:** 1 Department of Community Nutrition, School of Nutritional Sciences and Dietetics, Tehran University of Medical Sciences (TUMS), P.O. Box 14155-6117, Tehran, Iran; 2 Department of Public Health, Spinghar Institute of Higher Education, Kabul Campus, Kabul, Afghanistan; 3 Spinghar Institute of Higher Education, Kabul Campus, Kabul, Afghanistan; 4 World FoodProgram, KIC, Kabul, Afghanistan; 5 Centre for Intelligent Healthcare, Coventry University, Coventry, CV1 5FB UK

**Keywords:** Diseases, Medical research

## Abstract

Metabolically healthy obesity (MHO) and metabolically unhealthy obesity (MUHO) are known to be affected by diet and inflammatory factors (such as TGF-β1, IL-β1, MCP1). We sought to survey that consume of processed meat could effect on MHO and MUHO phenotypes, mediated through inflammatory markers, in overweight and obese Iranian women. The current cross-sectional study was done on 224 women 18–48 years, with a body mass index (BMI) ≥ 25 kg/m^2^. A 147- item food frequency questionnaire (FFQ) was used to evaluate dietary intake. In all participants, anthropometric indices and biochemical factors, as well as metabolic health phenotypes based on Karelis score, were evaluated. According to results, 22.6% of participants had MHO and 75.7% had MUHO phenotypes. There was an association between higher adherence to processed meats and increasing odds of MUHO phenotype in Iranian women (OR:2.54; 95% CI 0.009, 7.51; *P* = 0.05). Moreover, we found that the relation can be affected by agents such as TGF-β1, IL-β1, and MCP1; however, more research is needed to confirm these results and finding.

## Introduction

Although obesity as a global health problem has metabolic disturbances, there are obese individuals who may have no associated pathologies. This group of individuals have metabolically healthy obesity (MHO) phenotype^1^. Moreover, metabolically unhealthy obesity (MUHO) phenotype are obese individuals with metabolic abnormalities, such as insulin resistance, hypertension^2–4^ or the presence of inflammation markers, such as C-reactive protein (CRP)^5,6^. The prevalence of MHO is very variable depending on the studies; however, it has been estimated to be 10%-30% worldwide^7^. MHO appears to be more prevalent in women, and even its prevalence decreases with age in both genders^8^. The underlying mechanisms causing the metabolic obesity phenotype are multiple and complex; however, we aimed to consider one of the prominent modifiable risk factors, which is diet compositions to reduce the prevalence of this phenotype.

It has been postulated that high-protein diets, specifically, contribute to weight gain or reduction, because they are often rich in red, and other products of, meat, such as processed meats^9,10^. Processed meats are rich in saturated fatty acid (SFA), cholesterol, sodium and nitrates leading to obesity, and chronic diseases such as diabetes and cancer^11,12^. It was shown that adherence to a healthy diet such as the consumption of fruits, vegetables, and whole grains is inversely related to chronic disorders (diabetes and CVD), whereas intake of processed meats or sugary drinks is related to raising risk^13–16^. Moreover, it has been posited that the consumption of fast food and organ meats could be associated with unhealthy phenotypes, even in people who were previously metabolically healthy in Iran^17^. Also, another study indicated the association of a western dietary pattern, consisting of red and processed meats, with obesity phenotype in adults^18^; whilst in Iranian men, it was found that the intake of meat may be associated with metabolically obese normal weight^19^. Also, an additional study showed that there could be a relationship between high red and processed meat consumption and insulin resistance and, therefore, MUHO^20,21^. There are limited studies investigating the association of processed meats and MHO phenotype, instead, they surveyed this relation with different dietary patterns^17,22,23^.

Furthermore, obesity and inflammation have a strong relationship leading to the inflammatory response triggered by excessive fat mass^24,25^. It was claimed that metabolically healthy obese and non-obese individuals had lower concentrations of inflammatory markers compared to their metabolically unhealthy counterparts^27,28^. Also, people with MUHO demonstrated significantly higher hs-CRP than with MHO^29^. Indeed, it has been shown that a higher intake of red and processed meat was significantly associated with higher C- reactive protein (CRP) in women^30^. To our knowledge, no study has investigated the extent to which inflammatory markers (TGF-β1, IL-β1, and MCP1) may mediate the relationship between processed meat and MUHO in women. This was the first study to investigate whether processed meat intake could influence MHO and MUHO phenotypes, considering Karelis criteria, mediated by inflammatory markers, in overweight and obese Iranian women. There are even fewer studies specifically worked on the association of processed meats and MUHO phenotype^31,32^. Thus, it is worthwhile to elucidate this relationship to be able to provide more knowledge and healthier lifestyle.

## Methods and materials

### Study population

This cross-sectional study was conducted using simple random sampling, where 224 women participants were recruited from 20 Tehran Health Centers. A total sample size of participants was determined by the this formula (([(Z1 − α + Z1 − β) × √1 − r^2^]/r)2 + ^2^), β:0.95, α:0.05, with 95% confidence and 80% power, and r:0.25)^33^. The inclusion criteria were; aged from 18 to 48 years old, with a body mass index (BMI) ≥ 25 kg/m^2^, lack history of hypertension, no intake of alcohol and opiate drugs, not being pregnant, not having an acute or chronic infection, and exclusion criteria were having a history of CVD, thyroid, cancer, diabetes, liver and kidney disease, and smoking. In addition, participants who had been following any arbitrary/special dietary regimen, as well as those with chronic disease(s) affecting their diet, or if their daily energy intake was < 800 kcal or > 4200 kcal^34^, were excluded. All participants were asked to provide written consent prior to study commencement. The study was approved by the ethics committee of Tehran University of Medical Sciences (R.TUMS.VCR.REC.1395.1234).

### Anthropometric measurements

Body composition, including fat and lean mass, and waist to hip ratio, was assessed by the bioelectric impedance analyzer (In Body 770 scanner, Korea)^35^. Weight was measured with an accuracy of 100 g using a Seca scale (made in Germany) with the least clothes and without shoes. Also, height was measured to the nearest 0.5 cm by non-elastic tape, subsequently, BMI was calculated as weight (kg) divided by height (m^2^). WC measurement was performed at the umbilicus, after exhalation, according to standard kin anthropometric guidelines. According to the World Health Organization (WHO) criteria for classification of weight, BMI 25–29.9 kg/m^2^ was considered as overweight, and BMI 30 kg/m^2^ or higher as obesity^36^.

### Biochemistry measurements

Blood samples were drawn after 12 h of overnight fasting to assess low-density lipoprotein (LDL-C), high-density lipoprotein (HDL-C), triglycerides (TG), fasting blood sugar (FBS), total cholesterol, Homeostatic model assessment insulin resistance (HOMA-IR), transforming growth factor-beta 1 (TGF-β1), interleukin- beta 1 (IL- β1), and monocyte chemoattractant protein-1 (MCP-1). The serum was separated and stored at a temperature of -70 °C until the analyses were carried out, after centrifuging.

### The HOMA-IR calculation

IR was calculated by the homeostatic model assessment (HOMA) according to the following equation: HOMA-IR ¼ [fasting plasma glucose (mmol/l) fasting plasma insulin (mIU/l)]/22.5^37^.

### Metabolic health phenotypes

According to Karelis criteria, the presence of three or more following items indicates metabolic phenotypes:: TG ≤ 1.7 mmol/L or use of lipid-lowering drugs, HDL-C ≥ 1.3 mmol/L, LDL-C ≤ 2.6 mmol/L, HOMA ≤ 2.7, and CRP ≤ 3.0 mg/L^38^. Then, participants were categorized into two groups, MHO and MUHO^39^.

### Dietary intake measurement

The dietary intake of the women was collected by expert nutritionists who conducted a face-to-face interview with the 147-item semi-quantitative food frequency questionnaire (FFQ), where its validity and reliability have been previously avowed in an Iranian population^40^. The average consumption frequency was considered over the past year on a daily, weekly, and monthly basis. Household measures were taken into account for portion sizes and then converted to grams^41^. The food composition table (FCT) of the United States Department of Agriculture (USDA) was used to evaluate energy and nutrients, although the Iranian FCT was considered for local foods that were not present in the USDA FCT. Processed meats were considered as sausage, hamburger, and salami. Moreover, total daily energy intake was examined by considering the sum of each food item's energy.

### Physical activity measurement

A valid and reliable international physical activity questionnaire (IPAQ), designed by the WHO and previously validated in Iranian women adults, was used to assess physical activity levels^42^. The participants were asked to answer questions, such as the time they spent walking, and moderate and vigorous physical activity, during the last week. Subsequently, the time of each physical activity was converted to minutes per week and calculated as the metabolic equivalent of the task (MET/minutes/week).

### Other covariates assessments

Demographic characteristics (including age, sex, income, marital status, supplements consumption, socioeconomic status, education, and occupation status) were collected. In addition, systolic and diastolic blood pressure (SBP and DBP) were evaluated after 15-min rest, using a mercury sphygmomanometer.

### Statistical analysis

Participants were categorized according to tertiles of processed meat consumption. The Kolmogorov—Smirnov test and histograms were used to determine the distribution of the data. All variables with normal distribution were analyzed by parametric tests. The one-way analysis of variance (ANOVA) for continuous variables and the chi-square test for categorical variables were used to compare subject characteristics and dietary intake across tertiles of the processed meat score, and they were reported as mean (SD). Analysis of covariance (ANCOVA) was used to examine demographic characteristics, anthropometric measurements, clinical assessments, and dietary intake across tertiles of the processed meat score via adjusting for age, BMI, physical activity, energy intake, economic status, and supplement consumption. To examine the association between processed meat score and MHO and MUHO, the binary logistic regression model was applied as an odds ratio (OR) and 95% confidence interval (CI). The Barrett method, Mediation Analysis Model, was implemented for the assessment of the mediating effects of TGF-β1, IL-β1, and MCP1, separately. In the current study, SPSS software version 26 (Chicago—United State) was used for data analysis, whilst a *P*-value < 0.05 was, a priori, considered statistically significant.

### Ethics approval and consent to participate

Each participant was informed completely regarding the study protocol and provided a written and informed consent form before taking part in the study. This study was conducted according to the declaration of Helsinki and all procedures involving human patients were approved by the TUMS. The study protocol was approved by the ethics committee of Tehran University of Medical Sciences (TUMS) with the following identification (R.TUMS.VCR.REC.1395.1234.).

## Results

### Study population characteristics

In total, 224 women, consisting of 53 MHO (22.6%) and 178 MUHO individuals (75.7%), with mean age and BMI of 36.2 years old and 30.5 kg/m^2^, respectively, participated in the study. The mean (SD) of MCP1, TGF, and IL-β1 were 52.6 (16.4) pg/ml, 79.4 (66.0) pg/ml, and 2.7 (2.6) pg/ml, respectively.

### General characteristics of participants across the tertiles of processed meat scores

General characteristics of participants across the tertiles of processed meat scores in overweight and obese women are shown in Table1. According to the crude model, participants with a lower score of processed meat were more physically active (*P* = 0.04); however, there were no significant differences between tertiles in other characteristics. After controlling for age, physical activity, and energy intake, we found that socioeconomic status (*P* = 0.01), supplement use (*P* = 0.03), LDL-C (*P* = 0.05), and HDL-C (*P* = 0.01) had statistically significant mean differences.

**Table 1:** General characteristics of participants across tertiles of processed meat (n = 224).

	T1 (n = 80)	T2 (n = 77)	T3 (n = 67)	*P*-value	*P*-value*
Age (year)	36.21 ± 7.64	36.54 ± 9.04	35.83 ± 9.55	0.88	–
Physical activity (MET/h/w)	1258.16 ± 1500.60	782.49 ± 742.10	948.43 ± 995.59	**0.04**	**–**
Anthropometric measurements					
Weight (kg)	80.17 ± 11.13	79.30 ± 10.00	78.56 ± 10.35	0.65	0.53
Height (cm)	161.41 ± 5.81	161.05 ± 6.01	161.32 ± 5.21	0.91	0.65
BMI (kg/m^2^)	30.70 ± 3.68	30.70 ± 3.44	30.30 ± 3.68	0.75	0.33
WHR	0.93 ± 0.05	0.93 ± 0.04	0.92 ± 0.05	0.83	0.3
Inflammatory markers					
TGF-β1 (pg/ml)	73.78 ± 33.99	79.90 ± 38.38	84.34 ± 73.04	0.55	0.66
IL-β1 (pg/ml)	2.73 ± 0.86	2.59 ± 0.94	2.93 ± 1.04	0.48	0.34
MCP1 (pg/ml)	52.04 ± 76.69	57.63 ± 113.64	53.51 ± 99.92	0.94	0.36
Blood pressure measurements					
SBP (mm/Hg)	113.41 ± 14.85	110.66 ± 13.39	109.80 ± 13.00	0.26	0.34
DBP (mm/Hg)	78.02 ± 10.99	78.53 ± 9.58	76.85 ± 9.35	0.59	0.71
Biochemistry assessments					
TG (mmol/L)	1.39 ± 0.79	1.41 ± 0.78	1.38 ± 0.81	0.96	0.6
^c^LDL-C (mmol/L)	2.43 ± 0.53	2.45 ± 0.67	2.48 ± 0.67	0.9	**0.05**
^c^HDL-C (mmol/L)	1.15 ± 0.27	1.18 ± 0.27	1.24 ± 0.26	0.12	**0.01**
Total cholesterol (mmol/L)	4.69 ± 0.82	4.80 ± 1.00	4.88 ± 0.97	0.47	0.24
FBS (mmol/L)	4.80 ± 0.53	4.93 ± 0.58	4.85 ± 0.52	0.3	0.4
HOMA-IR	3.23 ± 1.30	3.65 ± 1.28	3.31 ± 1.26	0.1	0.12
^¥^Socioeconomic status				0.06	**0.01**
Low	23 (40.0)	20 (34.5)	16 (25.5)		
Medium	39 (38.1)	36 (35.1)	28 (26.8)		
High	18 (28.8)	21 (33.9)	23 (37.3)		
^¥^Occupation status				0.37	0.45
Unemployed	2 (50.0)	2 (50.0)	0 (0.0)		
Employed	78 (35.5)	75 (34.1)	67 (30.5)		
^¥^Marital status				0.17	0.84
Single	21 (32.3)	19 (32.3)	23 (35.5)		
Married	59 (36.6)	58 (36.0)	44 (27.3)		
^¥^Education status				0.2	0.83
Low	9 (30.8)	8 (26.9)	13 (42.3)		
Diploma	34 (38.6)	30 (34.1)	24 (27.3)		
High	37 (34.9)	39 (36.8)	30 (28.3)		
^¥^Supplement use				0.1	**0.03**
Yes	52 (39.6)	44 (32.1)	40 (28.3)		
No	28 (28.6)	33 (37.1)	27 (34.3)		

BMI, body mass index; DBP, diastolic blood pressure; FBS, fasting blood sugar; FFM, Fat free mass; HDL, high density lipoprotein; IL- β1, interleukin- beta 1; LDL, low density lipoprotein; MCP1, monocyte chemoattractant protein-1; PDI, overall plant-based diet index; SBP, systolic blood pressure; TGF-β1, transforming growth factor beta 1; WHR, waist hip ratio.

Values are mean ± standard deviation (SD) for continuous variables and number, percentage for dichotomous variables.

Using one-way ANOVA for continuous variables and Chi-square test for categorical variables.

*P-value: adjusted for energy intake, age, physical activity.

^a^Significant mean difference between tertiles one and two.

^b^Significant mean difference between tertiles one and three.

^c^Significant mean difference between tertiles two and three.

^¥^The variables that were reported in n (%).

*P*-value < 0.05 significant.

### Dietary intake of participants across the tertiles of processed meat scores

Dietary intake of participants across the tertiles of processed meat scores in overweight and obese women are presented in Table 2. Before adjusting for confounding factors, energy intake, all macro-nutrients, as well as cholesterol, SFA, and monounsaturated fatty acid (MUFA) consumption in higher scores of processed meat were significantly lower than the first tertile (*P* < 0.001). Moreover, participants in the highest tertiles of processed meat had lower intake of linoleic acid, linolenic acid, sodium, potassium, iron, magnesium, calcium, zinc, selenium, vitamin A, β carotene, thiamin, vitamin B_6_, folate, vitamin B_12_, total fiber, refined grains, fruits, vegetables, nuts, dairy, and legumes (*P* < 0.001). Whereas, after controlling for energy intake, the higher consumption of processed meat was only associated with lower intake of magnesium (*P* = 0.00), total fiber (*P* = 0.01), and nuts (*P* = 0.05). However, other dietary factors across tertiles of processed meat showed no significant results before and after adjusting for energy intake.

**Table 2:** Dietary intake of participants across tertiles of processed meat.

	Mean ± SD			*P*-value	*P*-value*
	T1 (n = 80)	T2 (n = 78)	T3 (n = 67)		
Energy intake (kcal/d)	3062.19 ± 568.97	2272.26 ± 614.58	2359.73 ± 786.33	** < 0.001**	–
Macronutrients					
Protein (g/d)	104.10 ± 21.92	78.97 ± 28.04	79.76 ± 27.23	** < 0.001**	0.84
Carbohydrate (g/d)	444.52 ± 106.46	323.93 ± 94.59	329.41 ± 118.48	** < 0.001**	0.36
Total fat (g/d)	107.62 ± 26.18	80.74 ± 29.53	87.57 ± 32.52	** < 0.001**	0.42
Micronutrients					
Cholesterol (mg/d)	296.52 ± 111.14	224.82 ± 82.87	228.15 ± 89.98	** < 0.001**	0.63
SFA (g/d)	32.59 ± 11.28	23.76 ± 8.92	26.09 ± 11.29	** < 0.001**	0.76
MUFA (g/d)	34.61 ± 9.28	27.13 ± 11.09	29.45 ± 10.90	** < 0.001**	0.45
Linoleic acid (g/d)	18.58 ± 6.98	15.21 ± 8.56	16.50 ± 7.92	**0.025**	0.58
Linolenic acid (g/d)	1.48 ± 0.64	1.05 ± 0.56	1.09 ± 0.57	** < 0.001**	0.23
Trans fatty acid (g/d)	0.00 ± 0.00	0.00 ± 0.00	0.00 ± 0.00	0.23	0.35
Na (mg/d)	4803.63 ± 1333.23	3798.20 ± 1302.93	3913.71 ± 1287.54	** < 0.001**	0.93
K (mg/d)	5270.56 ± 1245.14	3847.06 ± 1435.79	3825.56 ± 1638.22	** < 0.001**	0.32
Fe (mg/d)	22.28 ± 4.89	16.28 ± 4.91	16.71 ± 6.09	** < 0.001**	0.54
Calcium (mg/d)	1381.84 ± 387.82	1038.43 ± 395.07	1020.04 ± 369.27	** < 0.001**	0.20
Mg (mg/d)	561.12 ± 113.99	405.97 ± 134.34	404.18 ± 143.71	** < 0.001**	**0.00**
Zn (mg/d)	15.62 ± 3.13	11.32 ± 3.85	11.69 ± 4.51	** < 0.001**	0.29
Selenium (mg/d)	136.47 ± 37.45	106.74 ± 40.42	111.88 ± 40.95	** < 0.001**	0.48
Vitamin A (RAE/d)	963.82 ± 441.71	696.23 ± 330.85	682.44 ± 398.52	** < 0.001**	0.29
β carotene	6776.13 ± 4265.37	4616.44 ± 2901.09	4498.84 ± 3379.99	** < 0.001**	0.14
Vitamin D (µg)	2.21 ± 1.89	1.81 ± 1.34	1.80 ± 1.39	0.18	0.80
Vitamin E (mg)	17.47 ± 6.52	16.03 ± 9.67	16.55 ± 9.19	0.55	0.26
Thiamin (mg)	2.37 ± 0.55	1.84 ± 0.57	1.88 ± 0.64	** < 0.001**	0.64
Vitamin B_6_ (mg)	2.55 ± 0.56	1.95 ± 0.67	1.93 ± 0.74	** < 0.001**	0.51
Folate (µg/d)	699.16 ± 153.46	543.19 ± 151.06	551.31 ± 177.90	** < 0.001**	0.87
Vitamin B_12_ (µg/d)	5.05 ± 2.33	4.00 ± 2.11	4.04 ± 2.33	**0.00**	0.76
Total fiber (g/d)	55.58 ± 18.53	38.34 ± 13.20	38.39 ± 17.61	** < 0.001**	**0.01**

MUFA, mono unsaturated fatty acid; SFA, saturated fatty acid.

Values are mean ± standard deviation (SD) for continuous variables.

Using one-way ANOVA for continuous variables and Chi-square test for categorical variables.

*Adjusted for energy intake.

*P*-value < 0.05 significant.

### Association of MHO and MUHO phenotype across the tertiles of processed meat scores

The association of MHO and MUHO phenotypes across the tertiles of processed meat scores in overweight and obese women is shown in Table 3. There were no significant associations between MUHO phenotypes with the tertiles of processed meat scores in the crude model (*P* = 0.26), whilst after adjusting for age, BMI, physical activity, energy intake, women who were in the third tertiles displayed an increased odds ratio but was not statistically significant (*P* = 0.57). Whereas, after adjustment for age, BMI, physical activity, energy intake, economic status, and supplement consumption, there were increasing odds of MUHO in the third tertile compared to first tertile of processed meat (OR:2.54; 95% CI 0.009, 7.51; *P* = 0.05). This demonstrated that women with higher scores of processed meats had higher odds of MUHO; however, there was no significant *P*-trend for this relationship.

**Table 3:** Association of metabolic healthy and unhealthy phenotypes with processed meat in overweight and obese women (n = 224).

	T1 (n = 80)	T2 (n = 77)		*P*- value	T3 (n = 67)		*P*- value	*P*- trend
		OR	95% CI		OR	95% CI		
MUHO								
Crude model	1	1.10	0.52, 2.31	0.79	1.57	0.71, 3.48	0.26	0.83
Model 1	1	0.86	0.32, 2.27	0.76	1.29	0.52, 3.19	0.57	0.85
Model 2	1	0.80	0.26, 2.40	0.69	2.54	0.009, 7.51	**0.05**	0.97

CI, confidence interval; MUHO, metabolically unhealthy obesity; OR, odds ratio.

Reference group: Metabolic healthy obesity.

Reference group: First tertiles.

Binary logistic regression was used.

Model 1: adjusted for age, BMI, physical activity, energy intake.

Model 2: adjusted for model 2 further with economic status, supplement consumption.

*P*-value < 0.05 significant.

### Association of MUHO with processed meat scores mediated by inflammatory markers

Association of MHO and MUHO phenotypes across the tertiles of processed meat scores, mediated by inflammatory markers, in overweight and obese women is presented in Table 4. According to Barret model for assessment of mediation effects, three inflammatory markers, including TGF-β1, IL-β1, and MCP1, were included in the models of adjustment. Significant odds of MUHO across the third tertiles of processed meat were attenuated in the inflammatory markers adjusted model. This likely indicates that there was mediator effectiveness of TGF-β1 (*P* = 0.14), IL-β1 (*P* = 0.12), and MCP1 (*P* = 0.48), with increasing odds of MUHO among processed meat tertiles.

**Table 4:** Association of MHO and MUHO phenotypes across the tertiles of processed meat scores mediated by inflammatory markers in overweight and obese women (n = 224).

	Inflammatory markers	T1 (n = 80)	T2 (n = 77)		T3 (n = 67)		*P*-value
			OR	95% CI	OR	95% CI	
MUHO	^£^TGF-β1	1	0.61	0.13, 2.72	2.20	0.55, 8.80	0.14
	^€^IL-β1	1	0.36	0.03, 4.14	15.19	1.18, 194.60	0.12
	^¥^MCP1	1	0.48	0.14, 1.63	2.75	0.84, 8.97	0.48

CI, confidence interval; IL- β1, interleukin- beta 1; MCP1, monocyte chemoattractant protein-1; TGF-β1, transforming growth factor beta 1.

Reference group: Metabolic healthy obesity.

Reference group: First tertiles.

Linear regression was used.

Reference group: Metabolic healthy obesity.

^£^Adjusted with age, BMI, physical activity, energy intake, economic status, supplement consumption, and TGF-β1.

^€^Adjusted with age, BMI, physical activity, energy intake, economic status, supplement consumption, and IL-β1.

^¥^Adjusted with age, BMI, physical activity, energy intake, economic status, supplement consumption, and MCP1.

## Discussion

In this cross-sectional study, we found that in women with a higher score of processed meat intake, higher odds of MUHO were evident. Moreover, these associations were independent of age, BMI, physical activity, energy intake, thyroid, economic status, and supplement consumption. Additionally, three inflammatory markers, including TGF-β1, IL-β1, and MCP1, were included in the adjusted models.

Participants with higher consumption of processed meats had 2.54 times higher odds of increased MUHO. Processed meats are associated with increased risk of chronic diseases (such as cancer and diabetes) because they have a high content of nitrates, cholesterol, SFA, cholesterol and sodium^43–45^. This result is concordant with many studies. In the American population, individuals with unhealthy and overweight showed significantly higher intake of red meat, processed meat and fried foods when compared with those with metabolically healthy and normal weight^46–48^. In a study by Pereira et al., unhealthy pattern was positively associated with the metabolically healthy and metabolically unhealthy and overweight phenotypes in the fourth quartile and in the third and fourth quartiles of consumption^22^. The cross-sectional study among 6964 women showed that metabolically unhealthy but normal-weight subjects had a significantly greater consumption of processed meat, red meat, and fried foods, compared to women who were metabolically healthy and normal weight^49^.The Soltani et al. study showed a direct association between greater adherence to a pro-inflammatory diet and increase odds of unhealthy phenotype, high FBS, and also a relation to low-HDL-C levels in overweight and obese participants^23^. There was a relation between more consumption of dairy products, poultry, apples/pears, citrus fruits, magnesium, and tea/coffee and diminished risk of developing an unhealthy phenotype, but greater consumption of organ meats, potatoes, and fast foods can raise the risk of metabolic obesity^50^. A cohort study illustrated that metabolically healthy obesity was related to the risk of an unhealthy diet, and also there was a relation between high consumption of processed meat and raising the risk of unhealthy phenotype obesity, there was a relationship between more energy-content food patterns and high adherence to an unhealthy diet and raising risks such as metabolic disturbance and obesity^51–55^. Intake of red and processed meats also decreased with the increaseing tertiles, as reported in previous studies an unhealthy diet is related to a higher odds of metabolically unhealthy, which includes consuming more solid fats, red meat are known^39^. In a study that examined the association between a healthy plant-based diet and metabolic obesity phenotype with the mediating role of inflammatory factors such as TGF-β1, IL-β1, and MCP1, more adherence to healthful plant-based diet index had a considerably lower risk for the MUHO phenotype in women^56^. Increases in circulating inflammatory markers, such as transforming TGF-β1, IL- β1, and MCP-1, are known to contribute to metabolic diseases^26^. In Iranian men, it was found that it is possible that higher meat consumption was related to metabolically obese normal weight^57^.

Overall, the intake of meat is related to increase plasma concentration of iron that may contribute to the enhancement of oxidative stress and inflammatory mediators^58^. Also, it contains a high level of advanced glycation end products that are produced during the food preparation process^59,60^. Advanced glycation end products have pro-inflammatory functions, and increased inflammatory mediators can increase insulin resistance in tissues^61^. Whereas intake of healthy diet has been shown to be a direct relation with the MHO phenotype^62^. The SFAs in processed meats, leading to decreased insulin sensitivity^63^. Indeed, it was claimed that metabolically healthy obese and non-obese individuals had lower concentrations of inflammatory markers, compared to their metabolically unhealthy counterparts^27,28^. In addition, participants with MUHO demonstrated significantly higher hs-CRP than MHO^29^.

Our study includes several strengths, such as the use of different quality assurance and quality control strategies, and the use of an FFQ developed and validated for the population assessed. As well, to our knowledge, this represents the first investigation into the association between processed meat consumption and healthy and unhealthy metabolic phenotype obesity among Iranian women. Among the limitations, information bias cannot be ruled out since, as already pointed out, the under-or over-reporting of foods composing the dietary patterns, especially among obese individuals, may have contributed to the positive associations observed. Additionally, the relatively small sample size should be addressed in the future.

## Conclusion

We found that women with higher consumption of processed meat had higher odds of MUHO. Prospective and interventional studies in both genders, different populations and ethnicities need to be conducted to further the knowledge about examin inflammatory markers (TGF-β1, IL-β1, and MCP1) may mediate the relationship between processed meat and MUHO.

## Data Availability

The all authors declare that the data supporting the results of this study are provided in present article, and all the data in the present study will be available with the opinion of the corresponding author.

## Abbreviations

BMI: Body mass index
DBP: Diastolic blood pressure
FBS: Fasting blood sugar
HDL: High density lipoprotein
IL- β1: Interleukin-beta 1
LDL: Low density lipoprotein
MCP1: Monocyte chemoattractant protein-1
SBP: Systolic blood pressure
TGF-β1: Transforming growth factor beta 1
WHR: Waist hip ratio
MUFA: Mono unsaturated fatty acid
PUFA: Poly saturated fatty acid
SFA: Saturated fatty acid
CI: Confidence interval
MUHO: Metabolically unhealthy obesity
OR: Odds ratio
MHO: Metabolic healthy obesity
WC: Waist circumference
DASH: Dietary approaches to stop hypertension
CRP: C-reactive protein
WHO: World Health Organization
HOMA-IR: Homeostatic model assessment-insulin resistance
FFQ: Food frequency questionnaire
FCT: Food composition table
TGF-β1: Transforming growth factor-beta 1
USDA: United States Department of Agriculture
IPAQ: Physical activity questionnaire
MetS: Metabolic syndrome
FFAs: Free fatty acids
NHANES: The National Health and Nutrition Examination Survey
